# Lack of Association Between Sex Hormones, MDSCs, LDGs and pDCs in Males and Females With Systemic Lupus Erythematosus

**DOI:** 10.3389/fimmu.2022.888501

**Published:** 2022-06-27

**Authors:** Jessica M. Jones, Frances Smith, Emily Littlejohn, Trine N. Jorgensen

**Affiliations:** ^1^ Cleveland Clinic Lerner College of Medicine of Case Western Reserve University School of Medicine, Cleveland, OH, United States; ^2^ Department of Inflammation and Immunity, Cleveland Clinic Foundation, Cleveland, OH, United States; ^3^ Department of Rheumatologic and Immunologic Disease, Orthopaedic and Rheumatologic Institute, Lupus Clinic, Cleveland Clinic Foundation, Cleveland, OH, United States

**Keywords:** systemic lupus erythematosus, plasmacytoid dendritic cells, TLR, sex, low density granulocytes, interferon, MDSC

## Abstract

Plasmacytoid dendritic cells (pDCs) and low-density granulocytes (LDGs) are interferon-alpha producing cells that create a pro-inflammatory response in Systemic Lupus Erythematosus (SLE) leading to auto antibody production and organ damage. Both pDCs and LDGs have been shown to be dysfunctional in patients with active SLE. Myeloid-derived suppressor cells (MDSCs) have the capacity to control T and B cell activation and differentiation, and have recently been identified as cells of interest in SLE as well. While not fully understood, previous studies have suggested that pDCs are regulated in part by both X chromosome inactivation and estradiol. Whether sex chromosomes or sex hormones regulate MDSCs and LDGs remain to be determined. We aimed to explore the relative role of sex and sex hormones on pDC, MDSC and LDG frequency and function in SLE patients. We recruited patients with SLE as defined by ACR or SLICC classification criteria and healthy controls in conjunction with the Cleveland Clinic Lupus Cohort and Clinical Research Unit. We analyzed serum sex hormone levels by ELISA, and frequencies of pDCs, MDSCs, and LDGs among PBMCs and serum cytokine levels by flow cytometry. PBMCs were further analyzed for expression of genes involved in or induced by toll-like receptor (TLR)7 or TLR9 stimulation. In all SLE patients, the serum estradiol/testosterone ratio and levels of granulocytic MDSCs and LDGs were increased, while levels of pDCs were decreased. Furthermore, pDCs from active SLE patients expressed lower levels of TLR7 and TLR9 and showed diminished production of TLR9-induced IFNα and TNFα as compared to healthy controls. LDGs from healthy controls and SLE patients expressed very low levels of TLR7 and TLR9 and largely failed to respond to TLR9 stimulation. Thus, regardless of sex and sex-hormone levels, frequencies of pDCs, MDSCs and LDGs, TLR7 and TLR9 expression, and TLR9-driven cytokine production were similarly altered in male and female SLE patients.

## Introduction

Type I interferon (IFN-I) production, along with dysregulated plasmacytoid dendritic cells (pDCs) and low density granulocytes (LDGs) have been strongly implicated in the pathogenesis of systemic lupus erythematosus (SLE). As such, SLE patients were recognized to have a distinct gene expression signature characterized by the upregulation of a number of interferon stimulated genes (ISGs) (1, 2), and an accumulation of IFNα-producing pDCs at sites of inflammation such as renal tissue in patients with active lupus nephritis (3, 4). LDGs also accumulate in SLE patients, produce IFN-I, and respond vigorously to IFN-I or IgG stimulation by undergoing netosis; a mechanism implicated as a main source of nuclear antigen (5, 6). Oppositely, myeloid-derived suppressor cells (MDSCs) represent inhibitory cells with the capacity to control immune-mediated diseases. MDSCs are subcategorized into granulocytic (G-MDSCs) and monocytic (M-MDSCs), based on their respective expression of Ly6G or Ly6C in mice and CD15 or CD14 in humans (7). Interestingly, IFN-I has been shown to enhance the elimination of G-MDSCs by promoting extracellular trap formation in a mouse lupus model, suggesting that IFN-I could drive the loss of protective suppressive activity of G-MDSCs in SLE (8).

Women are nine times more likely to develop SLE than men, yet it remains unknown if sex hormone levels associate with key inflammatory and cellular players driving this disorder. A role of sex in IFN-I production is supported by studies showing that TLR7-mediated IFNα production was higher among healthy females than males (9). Subsequent studies revealed that treatment with estradiol *in-vivo* also lead to higher TLR7- and TLR9-mediated IFNα production, both in mice and in post-menopausal healthy women (10), although the study did not clearly identify the cells responding to TLR7 and producing IFNα. The gene for TLR7 itself lies on the X chromosome and is independently associated with IFN-I production (11). Furthermore, while one X chromosome is typically inactivated in female cells, the *TLR7* gene has been shown to escape X-inactivation, leading to higher rates of transcription in females compared to males (12). Interestingly, a recent study used samples from a healthy cohort including transgender patients to independently explore sex hormone and X-chromosome copy number influence (11). In this study, a positive association was found between testosterone and the percentage of pDCs producing IFNα after TLR7 stimulation in the presence of one X chromosome, while a negative association was identified in the presence of two X chromosomes, suggesting that factors independent of *TLR7* expression levels are involved in this regulation. Finally, ligand-mediated activation of TLR7 and TLR9 initiates a signaling cascade in which a complex including IRAK4 and IRAK1 is formed leading to the phosphorylation of IRF7 and IRF5. It is noteworthy that *IRAK1* is also transcribed on the X chromosome, and that within T cells of SLE patients, IRAK1 has been found to be over-expressed in females compared to males (13). Furthermore, *IRF5*, but not *IRF7*, was found to be expressed more highly by pDCs from healthy females than from healthy males (14).

Based largely on studies within lupus-prone mouse models, we have previously hypothesized a regulatory link between pDCs and myeloid derived suppressor cells (MDSCs), driven in part by sex hormones (15). In lupus-prone male mice, MDSC frequencies are controlled by testosterone and the cells remain immunosuppressive as the mice age while in female mice the cells lose their abilities to inhibit lymphocyte activation and differentiation after puberty (16, 17), suggesting a role for estrogens (or inflammation) in differentiating MDSCs into more immunostimulatory cell subsets. MDSC subsets are typically identified by cell surface markers, however these are often shared with other myeloid cell populations. For example, human G-MDSCs share the majority of surface markers with LDGs (18) and are known to readily differentiate into mature neutrophils (19).

Given that females are more likely to develop SLE than males, and the strong implications of pDCs, LDGs, and MDSCs in SLE and lupus-like pathogenesis, it is surprising that relatively little research exists exploring pDC, LDG and MDSC frequencies and functions between males and females with SLE. Furthermore, the significance to which sex and sex hormones may control these cells in SLE is not understood. We here investigated the levels of MDSCs, LDGs and pDCs, the expression of IFN-I-induced genes, and the responsiveness to TLR7 or TLR9 stimulation by these cells as a function of sex hormone levels among males and females with SLE or healthy controls. Surprisingly, we found that between males and females with SLE, sex hormone levels correlated with neither pDCs, LDGs, nor MDSCs. Furthermore, we found that both pDCs and LDGs from SLE males and females presented with a reduced capacity to produce IFN-I and TNFα following TLR7 or TLR9 activation, suggesting that dysregulation of these cell subsets is independent of sex hormones and sex chromosomes.

## Materials and Methods

### Patients and Samples

Lupus patients enrolling into the Cleveland Clinic Lupus Registry were simultaneously approached for enrollment in the current study. A total of 76 Lupus patients were enrolled (see **Table 1**). Patients were excluded from the study if they were currently pregnant, or were taking hormone therapy for reasons other than birth control. All patients met Lupus classification criteria as defined by either 2019 ACR/EULAR or SLICC 2012 ACR criteria. Lupus disease activity was assessed by each patient’s treating physician based on the SLE Disease Activity Index (SLEDAI). Disease organ involvement was determined from physician report and SLEDAI features at the time of enrollment. Additionally, healthy males and females were recruited and enrolled into the study through the Cleveland Clinic Clinical Research Unit. Healthy controls were excluded if they reported an autoimmune disease diagnosis, were currently pregnant, were taking hormone therapy for reasons other than birth control, steroid use in the last 30 days, or if they were taking any immunomodulatory medication. A total of 19 healthy participants were enrolled (see **Table 1**). The study was approved and monitored by the Cleveland Clinic Institutional Review Board. Patient Characteristic data was collected by the Lupus Registry and stored on a secure Redcap database.

**Table 1:** Participant Characteristics.

	Active SLE Females	Inactive SLE Females	Healthy Control Females	Active SLE Males	Inactive SLE Males	Healthy Control Males
**Total #**	15	50	14	5	6	5
Age*	43.7 (19-70)	44.6 (20-74)	37.57 (22-53)	33.6 (26-51)	52 (26-70)	32 (25-42)
**Race: ^+^**						
*White/Caucasian:* *Black/African American:* *Asian:* *Hispanic:* *Other:*	7 (47%) 6 (40%) 2 (13%) 0 (0%) 0 (0%)	32 (64%) 14 (28%) 1 (2%) 0 (0%) 3 (6%)	11 (79%) 0 (0%) 2 (14%) 1 (7%) 0 (0%)	2 (40%) 2 (40%) 0 (0%) 0 (0%) 1 (20%)	5 (83%) 0 (0%) 0 (0%) 0 (0%) 1 (17%)	4 (80%) 0 (0%) 0 (0%) 1 (20%) 0 (0%)
SLEDAI*	7.3 (6-12)	2.0 (0-4)		7.2 (6-8)	1 (0-2)	
**Disease Involvement**						
*Neuro* *Renal* *Cutaneous* *Serositis* *Arthritis* *Alopecia* *Oral/Nasal Ulcers* *Cytopenia*	1 (7%) 2 (14%) 4 (27%) 1 (7%) 6 (40%) 4 (27%) 1 (7%) 6 (40%)	0 (0%) 9 (18%) 10 (20%) 2 (4%) 15 (30%) 11 (22%) 12 (24%) 16 (32%)		1 (20%) 3 (60%) 2 (40%) 0 (0%) 2 (40%) 1 (20%) 0 (0%) 0 (0%)	0 (0%) 1 (17%) 3 (50%) 1 (17%) 0 (0%) 0 (0%) 0 (0%) 3 (50%)	
Duration of Disease* (years)	7.1 (0-33)	7.8 (0-39)		3.2 (0-8)	6.7 (1-25)	
**Steroid use ^+^**	7 (47%)	14 (28%)		1 (20%)	1 (17%)	
*Months use in last year* ^*^	5.7 (1-12)	8.2 (0-12)		2	12	
*Daily dose (mg)* ^*^	17.8 (5-40)	9.8 (2-30)		20	5	
**HCQ use ^+^**	12 (80%)	45 (90%)		5 (100%)	4 (67%)	
**NSAID use ^+^**	4 (27%)	17 (35%)		0 (0%)	2 (33%)	
**Methotrexate use ^+^**	1 (7%)	4 (8%)		0 (0%)	2 (33%)	
**Azathioprine use ^+^**	2 (13%)	6 (12%)		1 (20%)	2 (33%)	
**Mycophenolate mofetil use ^+^**	6 (40%)	12 (24%)		2 (40%)	0 (0%)	
**Leflunomide use ^+^**	1 (7%)	0 (0%)		0 (0%)	0 (0%)	
**Belimumab use ^+^**	1 (7%)	1 (2%)		0 (0%)	0 (0%)	

^*^ Mean (range). **^+^** Number of positive responses (percent).

Samples were processed within 4 hours of blood draw. For peripheral blood mononuclear cell (PBMC) isolation, blood samples were collected in EDTA coated tubes (BD biosciences, San Jose, CA), and spun down and plasma was collected. Samples were then resuspended in DMEM, and density gradient separation was performed using Ficoll-paque™ plus (Cytiva, Marlborough, MA). Purified PBMCs were immediately resuspended in charcoal inactivated fetal bovine serum (FBS) with 10% DMSO and cryopreserved. PAXgene Blood RNA tubes were processed according to manufacturer protocol (PreAnalytix, Hombrechtikon, Switzerland), and serum was obtained from serum separating tubes by centrifugation (BD biosciences, San Jose, CA).

### Enzyme-Linked Immunosorbent Assay

Serum samples were thawed and simultaneously run for all determinations of sex hormone levels to reduce freeze/thaw cycles. ELISA assays were run according to manufacturer protocols for Estradiol (undiluted, sensitivity: 10pg/ml) (Abnova, Taipei, Taiwan), Testosterone (undiluted, sensitivity: 0.0083 ng/ml) (Abcam, Cambridge, UK), Progesterone (1:200 dilution, sensitivity 10pg/ml)) (Cayman Chemical, Ann Arbor, MI) and DHEA-S (1:200 dilution, sensitivity 90pg/ml)) (Life technologies, Carlsbad, CA). If needed, values below the range of detection were reported as the lowest value detectable.

### Flow Cytometry

PBMCs were thawed briefly in a 37°C water bath before washing with DMEM. Cells were then resuspended in phosphate buffered saline (PBS, pH 7.2) and stained for flow cytometry. For intracellular staining, cells were first stained with surface stains, then fixed and permeabilized with foxp3/transcription factor staining buffer (Invitrogen, Waltham, MA) before staining with intracellular stains. Flow cytometry for PBMCs was run on a BD LSR Fortessa™ flow cytometer (BD Biosciences, San Jose California, USA) and data were collected using BD FACSDiva™ software (BD Biosciences, San Jose California, USA). Data were analyzed using FlowJo Version 10 Software (FlowJo, Ashland, Oregon, USA). The following antibodies were used for staining: PE CF594-conjugated anti-CD15; APCH7-conjugated anti-CD14; PE-conjugated anti-CD33; APC-conjugated anti- HLA-DR, FITC-conjugated anti-Lineage; PE/Cy7-conjugated anti-CD123; PE-conjugated anti-TLR7 (all from BD Biosciences, San Jose, CA); A700-conjugated anti-CD11b (Life technologies, Carlsbad, CA); FITC-conjugated anti-TLR7 and FITC-conjugated anti-Rabbit IgG isotype control (both from Invitrogen, Waltham, MA); biotinylated-anti TLR9 (Abcam, Cambridge, UK); unconjugated anti-human FC block, PerCP/Cy5.5-conjugated Streptavidin; biotinylated anti-Mouse IgG2a and PE-conjugated anti-Mouse IgG1κ (all from eBioscience inc, Santa Clara, CA).

### RNA Isolation and RT-PCR

RNA was isolated from PAXgene Blood RNA tubes using the PaxGene RNA isolation kit (PreAnalytix, Hombrechtikon, Switzerland). RNA was quantified using nanodrop technology (Nanodrop ND-1000 Spectrophotometer, Thermo Fisher). Complementary DNA (cDNA) was made from 100ng of RNA using qScript™ DNA supermix (Quanta BioSciences, Gaithersburg, MD) and quantified using nanodrop technology and diluted for qPCR. qPCR was performed with 100ng cDNA using Taqman Primers and TaqMan™ Fast Advanced Master Mix (ThermoFisher Scientific, Waltham, MA) and run on a Step One Plus real time PCR system (Applied Biosciences, Foster City, CA, USA). All transcripts were analyzed using 18S expression as a control. The following Taqman™ primer sets (ThermoFisher Scientific, Waltham, MA) were used: IFI27 (Hs01086370_m1), IFI44L (Hs00199115_m1), IFIT1 (Hs00356631_g1), ISG15 (Hs00192713_m1), RSAD2 (Hs01057264_m1), SIGLEC1 (Hs00988063_m1), IRAK4 (Hs00211610_m1), IRF5 (Hs00158114_m1), IRF7 (Hs01014809_g1), 18S (Hs999999001_s1). Each gene’s expression was first normalized to housekeeping gene 18S. A single healthy control sample was then used as a reference sample and all other samples are reported as gene expression relative to that sample. An ISG score to represent the overall interferon-induced gene expression signature was also developed. The score represents the median relative expression among all six individual genes for each patient.

### LEGENDplex™ Multiplex Cytokine Analysis

From a subset of patients, serum samples were thawed and diluted according to manufacturer’s recommendations. Samples were plated and stained with cytometric beads for cytokines using a LEGENDplex™ multiplex custom panel (BioLegend, San Diego, CA) following the manufacturer’s protocol. The assays were run on a Cytek^®^ Aurora with the support of the Lerner Research Institute flow cytometry core (Cytek Biosciences, Fremont, CA).

### pDC TLR Agonist Activation Assay

PBMCs were thawed briefly in a 37°C water bath, washed in media (RPMI w/L-glutamine, 10% charcoal inactivated fetal bovine serum, 1% non-essential amino acids, and 1% penicillin/streptomyocin) and counted on a Beckman Coulter Cell Counter (Beckman Coulter, Brea, CA). 10^6 cells were plated per well and incubated at 37°C with 5% CO_2_ for 2 hours prior to stimulation. Cells were treated with 2µM ODN2216 (Miltenyi Biotec, Bergisch Gladbach, Germany), 2µM ORN R2336 (Miltenyi Biotec, Bergisch Gladbach, Germany), 2µM ORN R2336 control (Miltenyi Biotec, Bergisch Gladbach, Germany) or media, and returned to incubator. After 2 hours, Golgi Plug (BD biosciences, San Jose, CA) was added to each well, and cells were subsequently incubated for another 4 hours until they were processed for flow cytometry staining as described above. The following antibodies were used in addition to those listed above and were all obtained from Miltenyi Biotec unless otherwise noted (Miltenyi Biotec, Bergisch Gladbach, Germany). PE-conjugated anti-IFNα, PE-conjugated anti-IL-6, PE-conjugated anti-human IgG1, APC-conjugated anti-TNFα, APC-conjugated anti-IL-10, APC-conjugated anti-human IgG1, APC-vio770-conjugated anti-HLA-DR, PerCP Cy5.5-conjugated anti-CD14 (ebioscience inc, Santa Clara, CA).

### Statistical Analysis

All statistical analyses were performed in GraphPad Prism (La Jolla, CA, USA). Comparisons among greater than two groups were performed using ANOVA testing or the non-parametric test, Kruskal Wallis testing with Dunn’s multiple comparisons where data was non-normally distributed. X by Y comparisons were performed using pearson correlation coefficient when data was normally distributed. A non-linear log transformed fit was calculated where appropriate, and R value reported. All error bars in graphs represent the median and interquartile range, except where noted otherwise. Statistical significance is defined as p < 0.05.

## Results

### Frequencies of pDCs Decrease, While Frequencies of LDGs and G-MDSCs Increase in Males and Females With Active SLE

**Figure 1:**
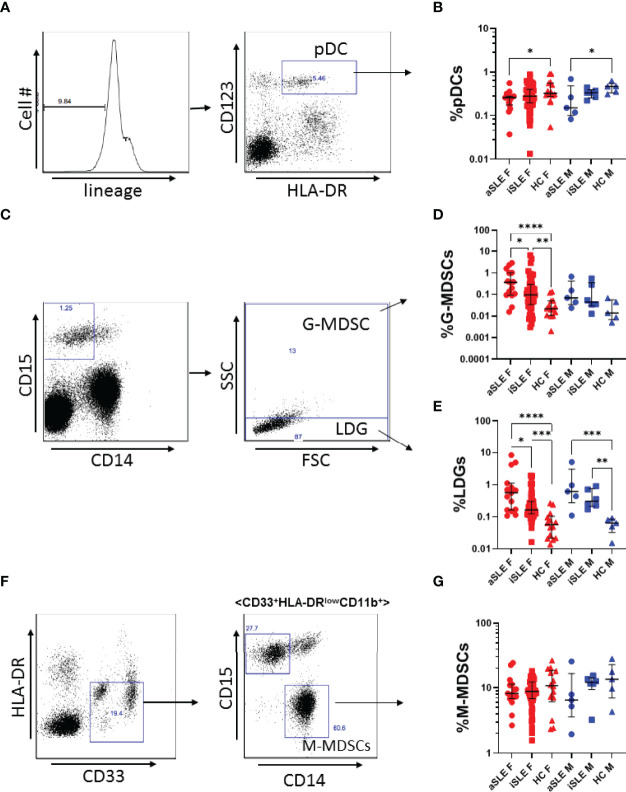
*Plasmacytoid dendritic cells are under expressed, while LDGs and G-MDSCs are overexpressed in SLE.* **(A)** Representative gating strategy for pDC identification. PBMCs were first gated as Lin^-^, and subsequently gated as CD123+, HLA-DR+. **(B)** pDC expression was decreased among males and females with active SLE. **(C)** Representative gating strategy for LDGs and G-MDSCs. LDGs were identified as CD15+CD14-SSC^low^, while G-MDSCs were identified as CD15+CD14-SSC^high^. **(D)** G-MDSCs and **(E)** LDGs were significantly increased among males and females with SLE. **(F)** Representative gating strategy for M-MDSCs. M-MDSCs were first identified as HLA-DR-, CD33+, and then gated for CD11b+, before gating as CD15-, CD14+. **(G)** M-MDSCs frequency was consistent among SLE patients and healthy controls. Comparisons were made using Kruskal-Wallis Test with Dunn’s test for multiple comparisons. Bars represent median and interquartile range. aSLE F (N = 15), iSLE F (N = 50), HC F (N = 14), aSLE M (N = 5), iSLE M (N = 6), HC M (N = 5). *p < 0.05; **p < 0.01; ***p < 0.001; ****p < 0.0001.

We first explored the frequency of pDCs, LDGs, and MDSCs among male and female patients with SLE and healthy controls by flow cytometry (**Figure 1**). Individuals with SLE were sub-classified as having active or inactive disease based on SLEDAI-2K scores. As there is currently no standard, we chose a score of 6 as the cutoff as this has been used by others (inactive: score < 6, active: score ≥ 6) (**Table 1**) (20). Within our population, both male and female patients with active SLE (and to a lesser extent inactive SLE) had decreased frequencies of pDCs compared to healthy controls (p < 0.05)(**Figures 1A, B**). Myeloid cell populations were identified simultaneously. LDGs were defined as CD15+CD14-SSC^low^ and G-MDSCs were defined as CD15+CD14-SSC^high^ (**Figure 1C**). M-MDSCs were defined as CD14+, CD15- (**Figure 1F**). All myeloid cell populations were also HLA-DR^low/neg^, CD33+ and CD11b+ with greater that 95% consistency (data not shown). LDGs and G-MDSCs were upregulated in both males and females with SLE (p < 0.01-0.0001)(**Figures 1D, E**). We found no differences in M-MDSC frequency between SLE patients and healthy controls, and further studies did therefore not include analyses of these cells (**Figure 1G**). There was no differences in the frequency of these cell types with increasing age among SLE patients or healthy controls, and among all groups the ratio of G-MDSCs to LDGs remained constant (data not shown). In contrast, ratios of pDCs to LDGs were positively correlated in healthy controls, while a negative association was observed in SLE patients (R=0.2 and -0.32 respectively) (**Supplemental Figure 1A**). This was true for both males and females. A similar, though non-significant trend was seen for frequencies of pDCs to G-MDSCs (R=0.07 and -0.18 respectively) (**Supplementary Figure 1B**).

### Patients *With* Systemic Lupus Erythematosus *Are Hormonally Dysregulated*

**Figure 2:**
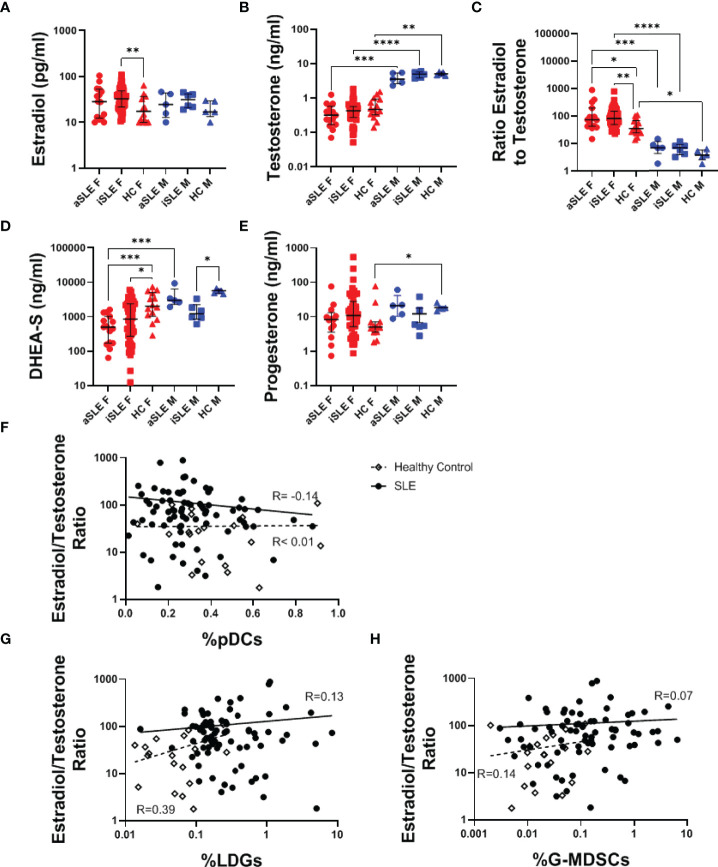
*Female SLE patients have distinct hormonal profiles.* Serum samples were analyzed *via* ELISA for levels of **(A)** Estradiol, **(B)** Testosterone, **(D)** DHEA-S, and **(E)** Progesterone. **(C)** The ratio of estradiol to testosterone was also analyzed and found to be increased among both males and females with SLE. Comparisons were made using Kruskal-Wallis Test with Dunn’s test for multiple comparisons. Bars represent median and interquartile range. The estradiol/testosterone ratio did not significantly correlate to expression of **(F)** pDCs, **(G)** LDGs, or **(H)** G-MDSCs. aSLE F (N = 15), iSLE F (N = 50), HC F (N = 14), aSLE M (N = 5), iSLE M (N = 6), HC M (N = 5). *p < 0.05; **p < 0.01; ***p < 0.001; ****p < 0.0001.

Given that women are nine times more likely to develop SLE than men, we explored the hormonal profiles of male and female SLE patients and healthy individuals. While no significant difference in estradiol or testosterone levels was identified between SLE patients and healthy controls (**Figures 2A, B**), the ratio of estradiol to testosterone was greater in both active SLE females and inactive SLE females compared to healthy females (p < 0.05-0.01)(**Figure 2C**). A similar, albeit not statistically significant, relationship was observed in the male cohorts (**Figure 2C**). It should be noted that within a multiple variable regression model of females only, the relationship between the estradiol/testosterone ratio and diagnosis of SLE remained statistically significant when controlling for the age of patients (p < 0.01) (data not shown). DHEA-S, a testosterone precursor molecule produced in the adrenal glands, decreased among all SLE patients compared to healthy controls (**Figure 2D**). Interestingly, while significant differences in serum progesterone were found among healthy males and females, this difference was lost among SLE patients (**Figure 2E**). Given these hormonal alterations, we further explored associations between hormonal levels and specific disease involvement. Among patients with SLE, females with renal involvement had a significantly higher estradiol/testosterone ratio than those without renal involvement (**Supplementary Figure 2A**), but no other significant associations were identified (**Supplementary Figures 2B–H**). There was also no significant correlation between the estradiol/testosterone ratio and SLEDAI (**Supplementary Figure 2I**). Finally, a non-significant negative association between pDC frequency and estradiol/testosterone ratio was found in SLE patients (**Figure 2F**, R= -0.14), while no such relationship existed in healthy controls. Oppositely, the frequencies of LDGs, and to a lesser extent G-MDSCs, correlated positively with estradiol/testosterone ratio (R= 0.39 and R= 0.14, respectively) in healthy individuals, but less so in SLE patients (**Figures 2G, H**).

### TLR Expression Is Down-Regulated in SLE pDCs

**Figure 3:**
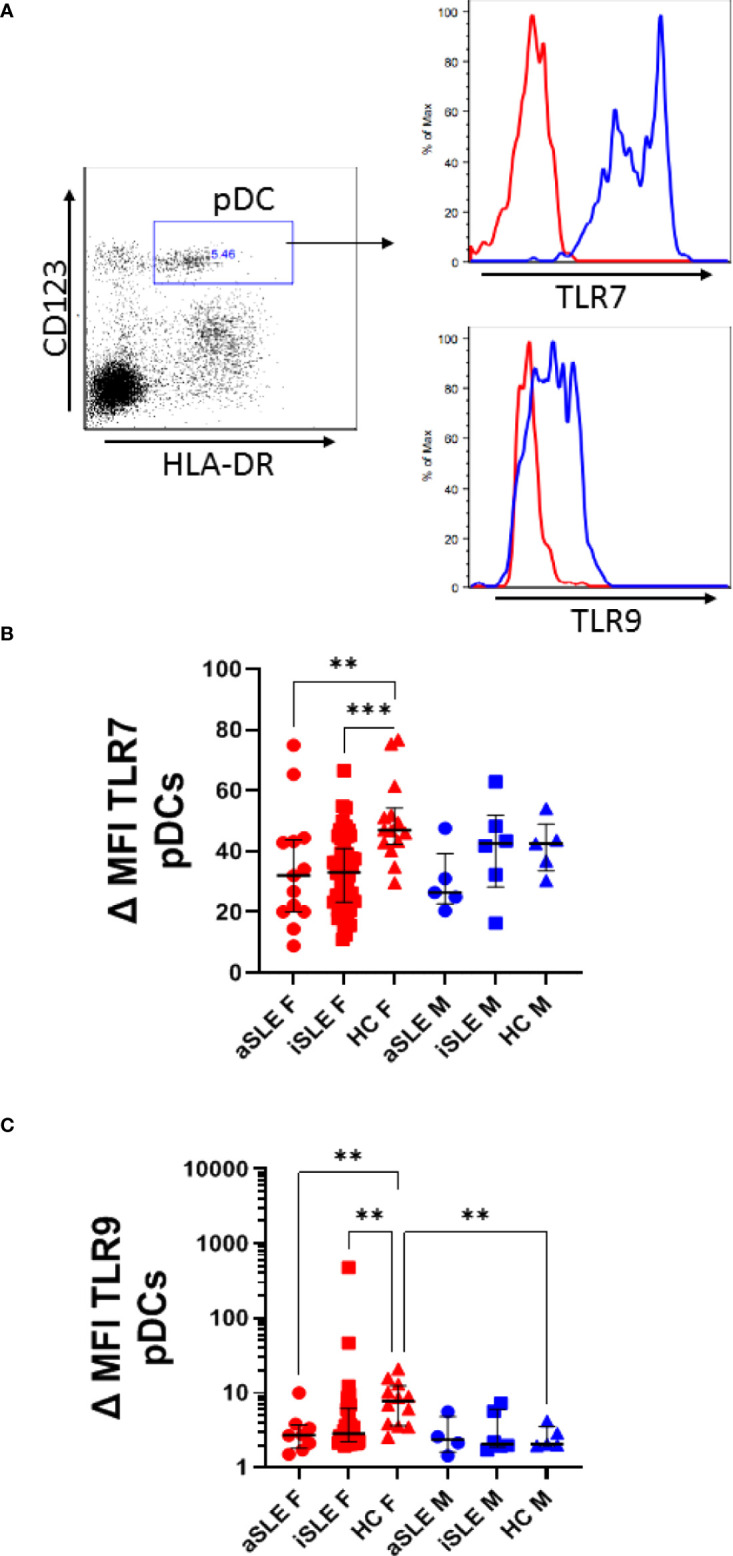
*TLR7 and TLR9 are downregulated among pDCs in SLE.* A subset of PBMCs were fixed and permeabilized and stained for intracellular TLR7 and TLR9 expression in pDCs. Mean fluorescent intensity was measured and normalized to isotype controls (shown in red) **(A)** SLE patients showed decreased change in MFI in **(B)** TLR7 and **(C)** TLR9. Comparisons were made using Kruskal-Wallis Test with Dunn’s test for multiple comparisons. Bars represent median and interquartile range. aSLE F (N = 8), iSLE F (N = 30), HC F (N = 12), aSLE M (N = 4), iSLE M (N = 6), and HC M (N = 5). **p < 0.01; ***p < 0.001.

Plasmacytoid DCs produce IFNα primarily through the activation of TLR7 and TLR9. pDCs from both male and female patients with active SLE displayed reduced TLR7 expression by pDCs compared to healthy individuals, although statistical significance was only apparent among females (p < 0.01) (**Figures 3A, B** and **Supplementary Figure 3A**). TLR9 expression was similarly decreased in female patients with SLE (p < 0.01) (**Figure 3C** and **Supplementary Figure 3B**). It should be noted that significantly lower levels of TLR9 expression was observed among healthy males compared to healthy females (p < 0.01), and that this difference was lost between males and females with SLE.

Although LDGs can produce IFNα, it is not known if this occurs *via* a TLR-dependent pathway. We therefore evaluated expression of TLR7 and TLR9 on LDGs but found negligible expression of both receptors (**Supplementary Figures 3C–F**). Ligation of TLR2 and TLR4 receptors can also stimulate IFNα production, prompting us to determine expression levels of these receptors as well. While TLR2 and TLR4 were expressed similarly between SLE patients and healthy controls, a slight trend towards higher levels of expression was observed in cells from active SLE patients (data not shown).

### *IRF7* Gene Expression, When Normalized to pDC Frequency, Remains Upregulated in SLE

**Figure 4:**
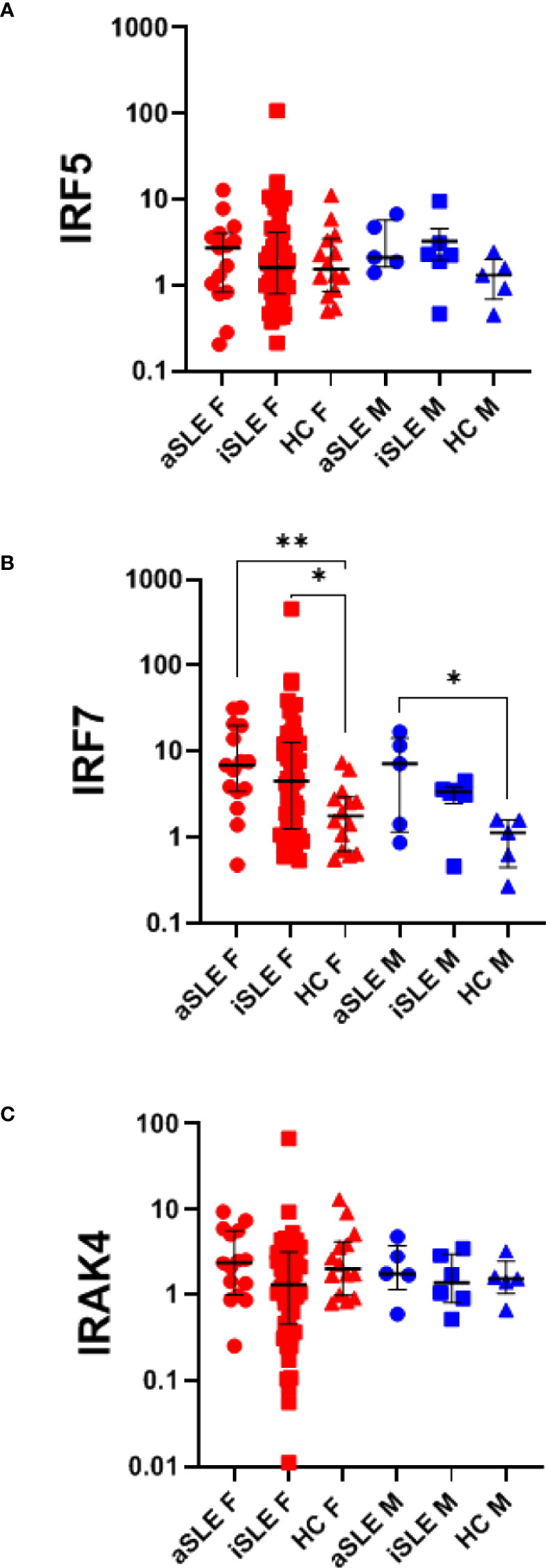
*IRF7 expression is upregulated in SLE, independent of pDC expression.* RNA was isolated from paxgene blood samples and RT-PCR was run using Taqman probes for signaling molecules within the TLR activation pathway: **(A)** *IRF5*, **(B)** *IRF7*, and **(C)** *IRAK4*. A single HC male sample was set as the standard level of expression and all samples were quantified as the relative gene expression to this control. Each sample was then normalized to its %pDCs as determined by flow cytometry analysis as presented in **Figure 1B**. Comparisons were made using Kruskal-Wallis Test with Dunn’s test for multiple comparisons. Bars represent median and interquartile range. aSLE F (N = 15), iSLE F (N = 50), HC F (N = 14), aSLE M (N = 5), iSLE M (N = 6), HC M (N = 5). *p < 0.05; **p < 0.01.

Given reports on TLR-induced IFNα production by SLE pDCs, and the downregulation of pDCs and their TLRs observed in our cohort of SLE patients, we evaluated if TLR7 and TLR9 activation pathways were affected by downstream signaling molecules in pDCs from SLE patients. Using purified whole blood RNA, we determined the relative gene expression of *IRAK4*, *IRF5* and *IRF7* by RT-PCR. *IRF7* gene expression was increased among males and females with SLE compared to healthy controls (p < 0.05-0.01), suggesting potential better responsiveness to TLR stimulation, while no differences were observed in expression levels of *IRAK4* and *IRF5* (**Figures 4A–C**). Although an association between *IRF5* (but not *IRF7*) and estradiol has previously been reported (14), we found no significant association between either *IRF5* or *IRF7* gene expression and estradiol levels in any of our samples (data not shown).

### Serum Levels of IFNα2 and TNFα Are Dysregulated in SLE Between Males and Females

Activation of IRF7 *via* phosphorylation and transport into the nucleus is required for IFNα gene expression, while concomitant activation of NF-κB drives transcription of pro-inflammatory cytokines including TNFα and IL-6 (21). In order to determine if any of these cytokines were differentially produced in SLE patients and healthy controls, we investigated the cytokine profiles of a subset of SLE patients and healthy controls. Patients included within this analysis were selected to represent a distribution of SLEDAI disease activity levels similar to the full sample set. In general, healthy control females showed higher levels of pro-inflammatory cytokines than healthy males did (**Table 2**). Moreover, both male and female SLE patients displayed slightly higher concentrations of IFNβ, IL-6 and IL-10 compared to healthy controls (**Table 2**). Interestingly, levels of IFNα2 and TNFα were closely correlated (R=0.95), and while females with SLE tended to have lower levels of these two cytokines than healthy control females, males showed the opposite trend, with SLE males expressing higher levels of IFNα2 and TNFα than healthy control males (**Table 2**).

**Table 2:** Cytokine profiles.

	aSLE F	iSLE F	HC F	aSLE M	iSLE M	HC M
**Number**	5	9	5	5	6	5
**IFNα2**	1.4 ± 1.2	6.2 ± 8.9	11 ± 20	3.3 ± 6.4	2.7 ± 6.3	1.9 ± 1.9
**IFNβ**	41 ± 13*	43 ± 55	18 ± 7.7	48 ± 25**	27 ± 9.2*	14 ± 4.5
**IL-6**	4.2 ± 6.6	1.9 ± 2.1	1.8 ± 2.5	2.5 ± 3.0	1.5 ± 2.6	0.6 ± 0.6
**IL-10**	4.3 ± 7.1	1.0 ± 0.9	2.2 ± 3.7	1.4 ± 2.8	1.5 ±2.7	0.5 ± 0.4
**TNFα**	1.1 ± 1.1	5.5 ± 5.0	12 ± 21	3.4 ± 6.9	4.1 ± 6.7	1.1 ± 0.8

Table 2, pDC associated cytokine expression is variable among SLE patients. A subset of serum samples were analyzed for cytokine expression using Legendplex bead assay following kit protocol. Data represents mean ± SD. *p value compared to sex-matched healthy control. *p < 0.05; **p < 0.01.

### Interferon Stimulating Genes (ISGs) Are Upregulated in Male and Female SLE

**Figure 5:**
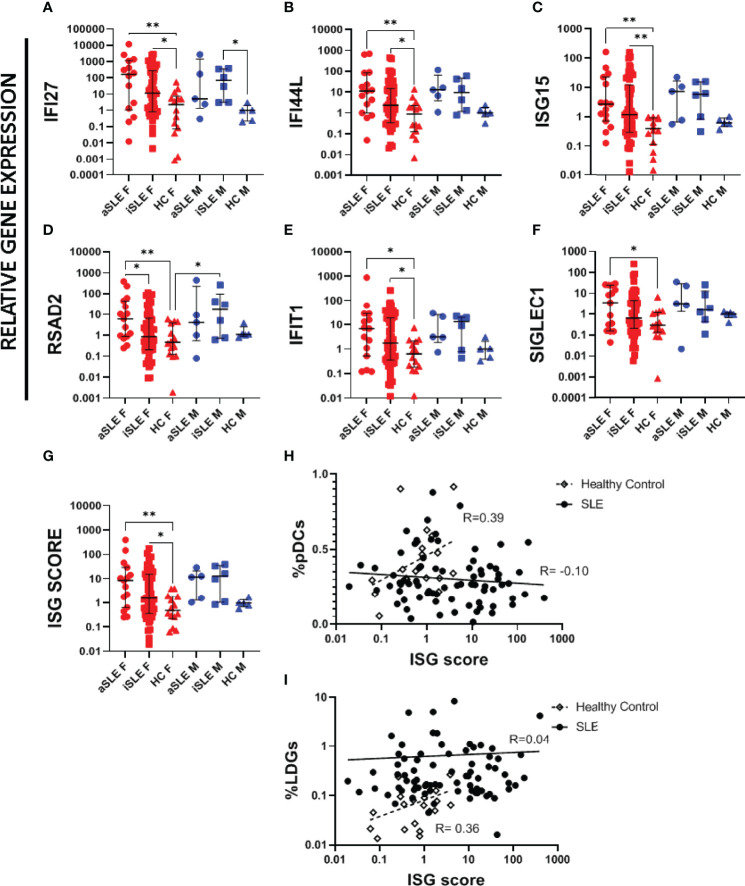
*Interferon stimulating genes are upregulated in SLE patients.* RNA was isolated from paxgene blood samples following kit protocol. RT-PCR was run using Taqman probes for known ISGs. A single HC male sample was set as the standard level of expression and all samples represent the relative gene expression to this control. The following ISGs were analyzed **(A)** *IFI27*, **(B)** *IFI44L*, **(C)** *ISG15*, **(D)** *RSAD2*, **(E)** *IFIT1*, F) *SIGLEC1*. **(G)** An overall ISG score was created by taking the median expression level of all 6 ISGs for each sample. ISG score was compared to **(H)** %pDCs and **(I)** %LDGs. Comparisons were made using Kruskal-Wallis Test with Dunn’s test for multiple comparisons. Bars represent median and interquartile range. ISG score was compared to individual pDC expression among healthy controls and patients with SLE, showing a loss of a positive correlation among SLE patients. aSLE F (N = 15), iSLE F (N = 50), HC F (N = 14), aSLE M (N = 5), iSLE M (N = 6), HC M (N = 5). *p < 0.05; **p < 0.01.

A limitation of serum cytokine analysis is that IFNα levels in serum fluctuate greatly within each individual. In contrast, evaluation of the expression level of interferon stimulated genes (ISGs) provide a representation of IFNα activity over an extended period. Using purified whole blood RNA, we investigated six different ISGs previously shown to be upregulated in SLE (22). In females, relative gene expression across all six genes increased stepwise from healthy controls to inactive SLE and to active SLE groups (**Figures 5A–F**). In males, gene expression levels between active and inactive SLE groups were more similar, and both appeared higher than their healthy control comparator group, although statistical significance was not reached due to the small sample size. An ISG score was developed by calculating the median expression among all six individual ISGs for each individual. Once again, female SLE patients displayed higher ISG scores than healthy female controls, while male SLE patients trended higher than healthy male controls (**Figure 5G**). Interestingly, the frequency of pDCs correlated positively with ISG scores among healthy controls (R=0.39)(**Figure 5H**), while this relationship was lost among patients with SLE (R=-0.10). Similarly, frequencies of LDGs correlated positively with the ISG scores among healthy controls (R=0.36), but was also lost among SLE patients (R=0.04)(**Figure 5I**). There was no significant association between ISG score and sex hormone levels for any of the groups (data not shown).

### pDCs From SLE Patients Are Poor Producers of IFNα Following TLR7 and TLR9 Stimulation

**Figure 6:**
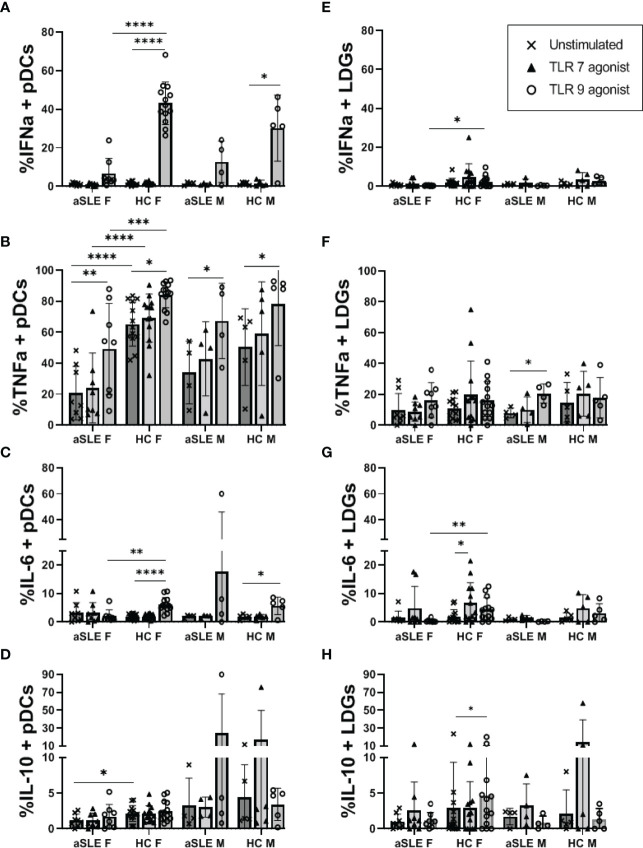
*TLR mediated production of IFNα and TNFα is decreased in patients with SLE.* PBMCs from a subset of healthy controls and patients with active SLE disease were cultured in the absence or presence of TLR7 agonist (ORN R2336) or TLR9 agonist (ODN2216) for 6 hours. Cells were then fixed and stained for intracellular levels of IFNα, TNFα, IL-6, and IL-10 in pDCs **(A–D)** and LDGs **(E–H)**. Comparisons were made by ANOVA with Fisher test for multiple comparisons. Bars represent mean and SD. aSLE F (N = 8), HC F (N = 13), aSLE M (N = 4), and HC M (N = 5). *p < 0.05; **p < 0.01; ***p < 0.001; ****p < 0.0001.

Finally, both pDCs and LDGs have been suggested as IFN-I producers in SLE. We cultured whole PBMCs from a subset of healthy control and SLE patients in the presence or absence of a TLR9 agonist (ODN2216), or a TLR7 agonist (ORN R2336), and measured the percentage of pDCs and LDGs producing IFNα, TNFα, IL-6 and/or IL-10. TLR9 agonist stimulation induced the accumulation of both IFNα and TNFα producing pDCs from healthy controls (**Figures 6A, B**). TLR9-stimulated pDCs from SLE patients also produced these cytokines, but to a significantly lesser degree and especially among females. It is of note that even with no stimulation, pDCs from healthy control females displayed increased TNFα production compared to pDCs from female SLE patients (p < 0.0001) (**Figure 6B**). Furthermore, though not statistically significant, pDCs from SLE males produced slightly more IFNα and TNFα than cells from SLE females (**Figures 6A, B**). Interestingly, in response to TLR9 ligation pDCs produced either TNFα alone, or IFNα and TNFα, but did not produce IFNα alone among any of our samples (**Supplementary Figure 4**). TLR9 stimulation mediated a small but significant increase in IL-6 production in healthy controls only (**Figure 6C**) and there were no significant changes to IL-10 production by TLR9-stimulated pDC in any of the samples (**Figure 6D**). LDGs were minimally stimulated by TLR9 stimulation (**Figures 6E–H**), only showing a small increase in IFNα and IL-6 production by LDGs of healthy females as compared to active SLE females (**Figures 6E, G**). None of the cells showed any consistent response to TLR7 agonist stimulation.

## Discussion

Despite evidence in the literature that sex chromosome copy number and sex hormones each have the capacity to promote the production of IFNα by pDCs more so in females than males, we saw few sex-associated differences within male and female SLE patients and healthy controls. While some early studies reported reduced testosterone levels in SLE patients (23), we found no significant differences in our cohort. It is possible that this discrepancy is due to our patient cohort’s low rates of steroid use, resulting in minimal effect on testosterone levels as previously observed (24, 25). This is supported by a sub-analysis of testosterone levels in female SLE patients only, which showed a significant decrease in testosterone levels in patients prescribed steroids (p < 0.001, data not shown).

The estradiol/testosterone ratio was increased in SLE patients, particularly in females, but also in males to a lesser extent. These results are consistent with prior studies, which showed increased aromatase activity in males and females with SLE leading to a higher estradiol/testosterone ratio that correlated with disease activity (23, 26). It is plausible that the altered estradiol/testosterone ratio among SLE males, in particular, creates a hormonal environment that is more “female like”. If that is correct, male SLE pDCs should function more similarly to female SLE pDCs. Indeed, while we found that healthy female pDCs produced more IFNα following TLR9 stimulation than healthy male pDCs, this difference was lost among male and female SLE patients, where the production of IFNα was uniformly low. Similarly, we found that pDCs from healthy females expressed more TLR9 compared to healthy males, and that this difference was also lost in males and females with SLE. Importantly, Seillet et al. found that estradiol increases the capacity of TLR9-mediated IFNα production in pDCs (10), which agrees with the findings we present in healthy individuals. Furthermore, it should be noted that prior studies have shown that estradiol promotes increased expression of some (but not all) pro-inflammatory cytokines in healthy controls, but not SLE patients (27–29). Our data support such dysregulation as healthy females had higher circulating levels of pro-inflammatory cytokines than healthy males did, while this difference was reduced between males and female SLE patients. Finally, in contrast to studies of healthy individuals, loss of TLR9 mediated cytokine production in SLE patients and patients identified as “at risk” for autoimmunity has been previously reported (30–32). Psarras et al. recently suggested that this may be due to functional exhaustion of pDCs in SLE disease and showed that pDCs from SLE patients displayed signs of cellular stress (32). Although speculative at this point, it is possible that an increased estradiol/testosterone ratio promotes activation of pDCs when the disease is developing at the cellular level, but prior to clinical identification, subsequently leading to an exhausted stage by the time of diagnosis. Large clinical studies following at-risk patients prior to full SLE disease development are needed to confirm this hypothesis.

Despite low levels of pDC-derived IFNα and TNFα production in our SLE patients, we still found that SLE patients displayed increased ISG expression similar to what have been extensively described in the literature (1, 2, 33, 34). Interestingly, we found that while the ISG score correlated with frequencies of pDCs in healthy controls, this relationship was lost among SLE patients. This may reflect a shift in the IFNα-producing cell(s) subset among SLE patients, or simply that IFNα-producing pDCs remain active despite being relocated to target tissues. In support of a shift in cellular origin, LDGs from SLE patients have been shown to produce IFNα *ex vivo*, and supernatants from PMA and G-CSF-stimulated LDGs can induce ISG expression within endothelial cell lines (35). Here, we found that LDGs were significantly increased in patients with active disease, and while pDCs and LDGs correlated positively among healthy controls, they were negatively correlated in SLE. In support of the pDC relocation hypothesis, however, we identified no association between ISG expression and LDG frequencies in SLE patients. Additionally, we found that LDGs are not activated *via* TLR7/9 pathways and identified no differences in LDG frequencies based on sex or sex hormones.

There are several limitations to our current study. First, as for most clinical studies, the small cohort size represents a significant limitation, especially within our male cohorts of both patients and controls. Additionally, our healthy control cohort under-represents African American individuals compared to our SLE cohort. This is particularly noteworthy given that race-related differences have been reported in SLE disease after attempting to control for social factors (36). Secondly, our patients were on a number of medications at the time of study enrollment, which potentially affected our findings. For example, the majority of patients in our study were taking hydroxychloroquine, which has been shown to impact TLR7/9 activation and reducing IFNα and TNFα production by pDCs in SLE patients (37, 38). Additionally, some of the patients were on systemic steroids, which could have affected the activity of pDCs. Guiducci et al. previously found that TLR7 and TLR9 activation antagonized the apoptotic actions of glucocorticoids on pDCs in SLE patients due to a lack of glucocorticoid activity on TLR-induced NF-κB activation (39). Preliminary analyses of our cohort of patients showed no difference in TLR9 mediated IFNα or TNFα production between SLE patients taking or not taking steroids (p = 0.63 and p = 0.94, respectively) or hydroxychloroquine (p = 0.52 and p = 0.25, respectively). Thirdly, G-MDSCs are closely phenotypically related to LDGs, only differing with regard to granularity. Both cells are known to be present within the ficoll lower density layer, with our isolation protocol likely capturing granulocytes of varying densities (7, 40). Although we have attempted to clearly distinguish between the two cell populations by gating for granularity, it remains a distinct possibility that a portion of the cells were misidentified. Thus, the identification of solid markers distinguishing these cell populations is needed to confirm our findings. Finally, another significant limitation relates to the observed minimal effect of TLR7 ligation on pDC activation. There are several plausible explanations: 1) We identified that even at baseline, a significant portion of pDCs expressed low levels of intracellular TNFα, which has been shown to inhibit the production of IFNα, and to a lesser extent TNFα, following TLR7 activation (41). This baseline TNFα production may offer an explanation of why we failed to see IFNα-positive cells, but still observed some TNFα induced following TLR7 activation. 2) We utilized charcoal inactivated FBS within our cell culture media to minimize the effects of exogenous sex hormones. It is however possible that TLR7 activation requires some level of extracellular estradiol to promote proper cytokine production. 3) It should also be noted that we only performed our experiment using ORN R2336 as a TLR7 agonist, following a protocol that mirrors what has recently been described in the literature to induce pDC activation (32). Whether we would have found the same results using another TLR7 agonist is unknown.

In conclusion, pDCs from male and female patients with active SLE uniformly displayed a decreased capacity to produce IFNα in response to TLR9 ligation. Interestingly, a dysfunctional TNFα response was only observed in females; an observation that requires further study. Since males and females with SLE often develop different signs and symptoms, and tend to have varying disease courses, other unidentified mechanisms mediated by sex or sex hormones may still differentially drive SLE in females versus males. Identification of such mechanisms requires further research and a commitment to enrolling and separately analyzing males and females within large cohort SLE studies.

## Data Availability Statement

The original contributions presented in the study are included in the article/**Supplementary Material**. Further inquiries can be directed to the corresponding author.

## Ethics Statement

The studies involving human participants were reviewed and approved by The Institutional Review Board, Cleveland Clinic Foundation (IRB #20-833). The patients/participants provided their written informed consent to participate in this study.

## Author Contributions

JJ contributed to experimental design, running experiments, analyzing data and writing the manuscript. FS contributed to running experiments and analyzing data for the manuscript. EL contributed to identification and enrollment of SLE subjects, advising on manuscript. TJ contributed to experimental design, data analysis and writing of the manuscript. All authors contributed to the article and approved the submitted version.

## Funding

This research was supported by the Rheumatology Research Foundation and support from the Lerner Research Institute.

## Conflict of Interest

The authors declare that the research was conducted in the absence of any commercial or financial relationships that could be construed as a potential conflict of interest.

## Publisher’s Note

All claims expressed in this article are solely those of the authors and do not necessarily represent those of their affiliated organizations, or those of the publisher, the editors and the reviewers. Any product that may be evaluated in this article, or claim that may be made by its manufacturer, is not guaranteed or endorsed by the publisher.
