# Supplementary material for: Lack of Association Between Sex Hormones, MDSCs, LDGs and pDCs in Males and Females With Systemic Lupus Erythematosus
Source: Front Immunol. 2022 Jun 27;13:888501. doi: 10.3389/fimmu.2022.888501 (PMC9271771; doi:10.3389/fimmu.2022.888501)
Supplement: Supplementary file 1 [file Presentation_1.pptx]

## Slide 1
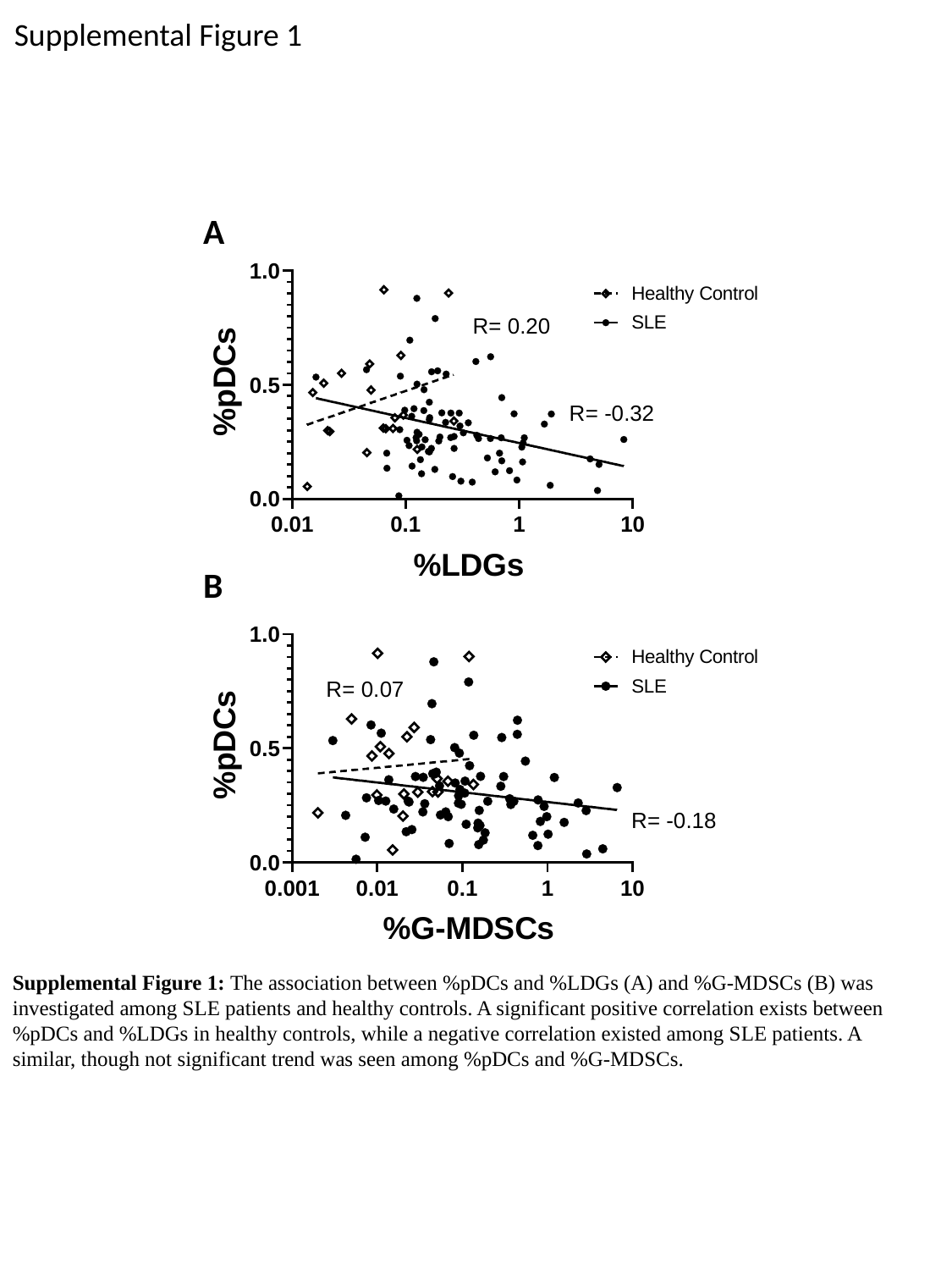

Supplemental Figure 1
A
B
Supplemental Figure 1: The association between %pDCs and %LDGs (A) and %G-MDSCs (B) was investigated among SLE patients and healthy controls. A significant positive correlation exists between %pDCs and %LDGs in healthy controls, while a negative correlation existed among SLE patients. A similar, though not significant trend was seen among %pDCs and %G-MDSCs.

## Slide 2
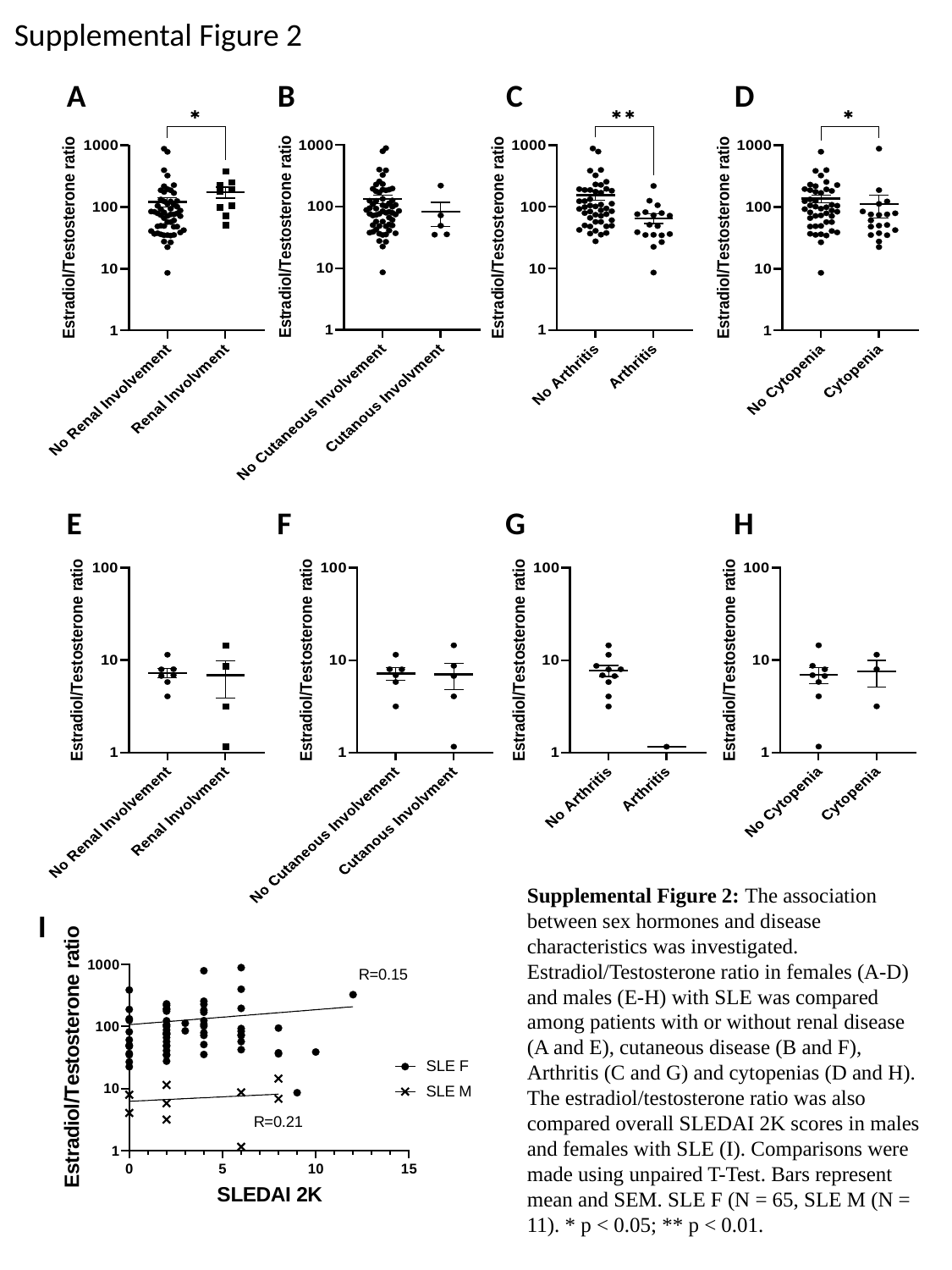

Supplemental Figure 2
A
B
C
D
E
F
G
H
I
Supplemental Figure 2: The association between sex hormones and disease characteristics was investigated. Estradiol/Testosterone ratio in females (A-D) and males (E-H) with SLE was compared among patients with or without renal disease (A and E), cutaneous disease (B and F), Arthritis (C and G) and cytopenias (D and H). The estradiol/testosterone ratio was also compared overall SLEDAI 2K scores in males and females with SLE (I). Comparisons were made using unpaired T-Test. Bars represent mean and SEM. SLE F (N = 65, SLE M (N = 11). * p < 0.05; ** p < 0.01.

## Slide 3
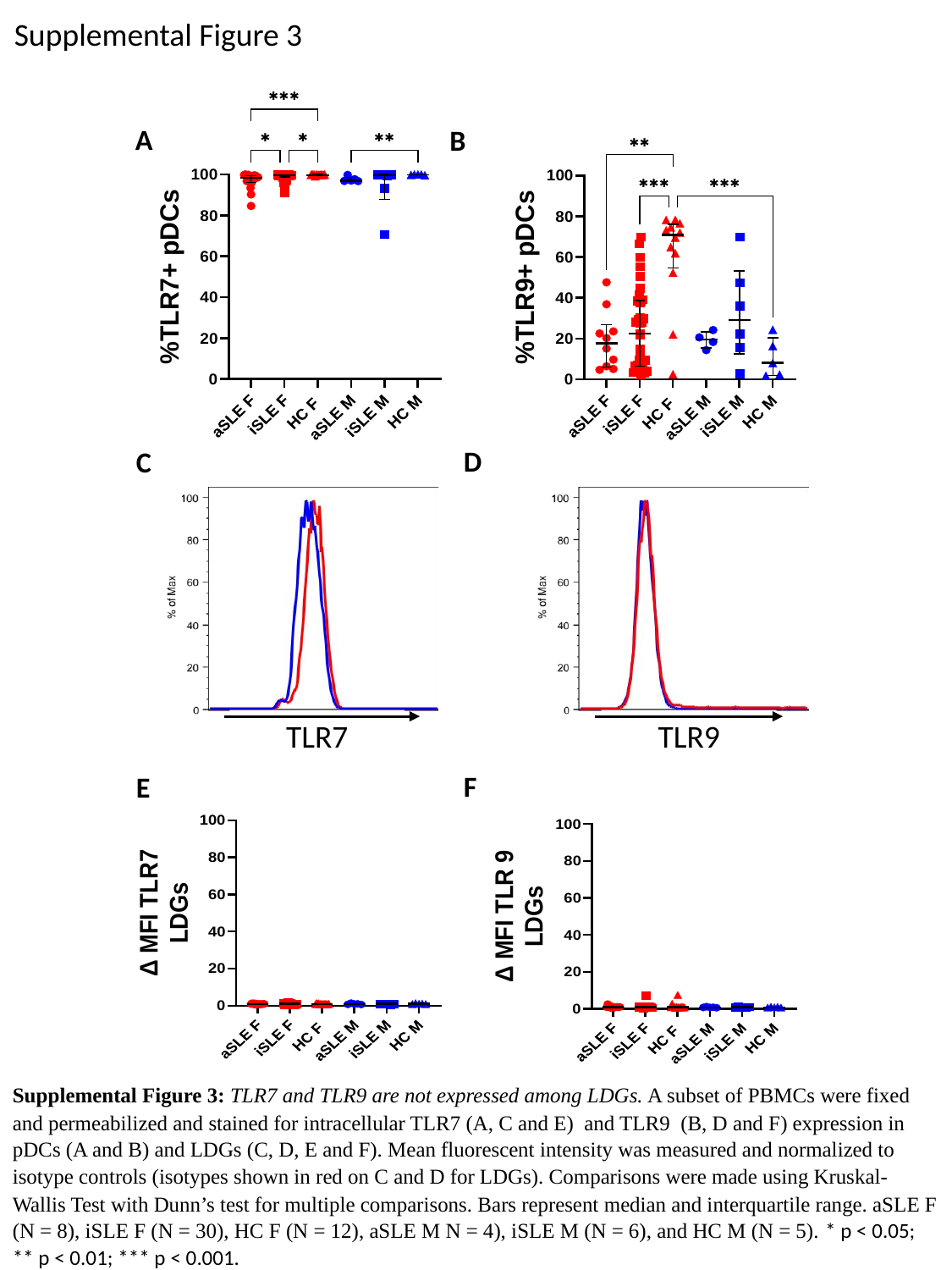

Supplemental Figure 3
A
B
D
C
TLR7
TLR9
F
E
Supplemental Figure 3: TLR7 and TLR9 are not expressed among LDGs. A subset of PBMCs were fixed and permeabilized and stained for intracellular TLR7 (A, C and E) and TLR9 (B, D and F) expression in pDCs (A and B) and LDGs (C, D, E and F). Mean fluorescent intensity was measured and normalized to isotype controls (isotypes shown in red on C and D for LDGs). Comparisons were made using Kruskal-Wallis Test with Dunn’s test for multiple comparisons. Bars represent median and interquartile range. aSLE F (N = 8), iSLE F (N = 30), HC F (N = 12), aSLE M N = 4), iSLE M (N = 6), and HC M (N = 5). * p < 0.05; ** p < 0.01; *** p < 0.001.

## Slide 4
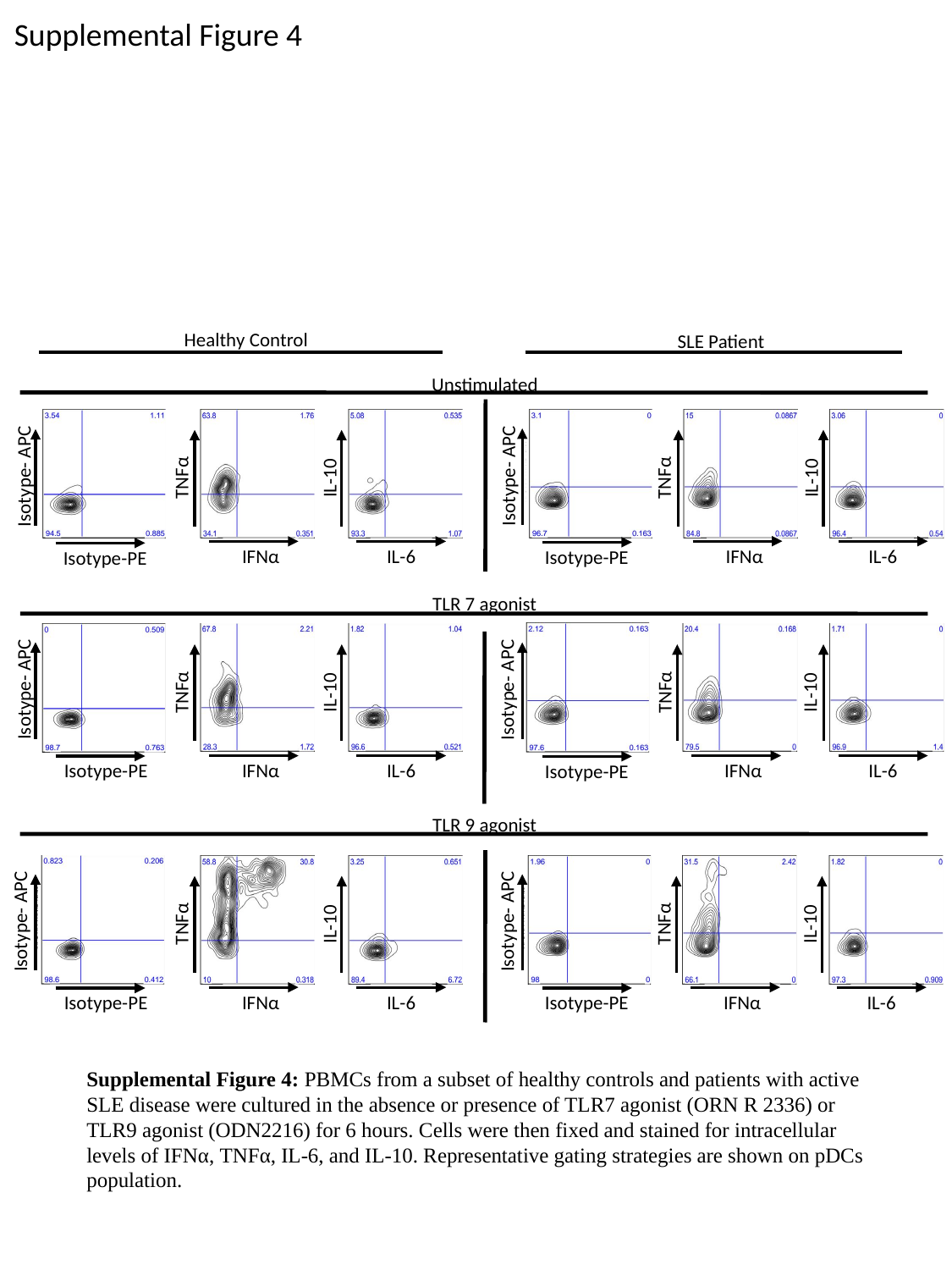

Supplemental Figure 4
Healthy Control
SLE Patient
Unstimulated
Isotype- APC
Isotype-PE
TNFα
IFNα
IL-10
IL-6
Isotype- APC
Isotype-PE
TNFα
IFNα
IL-10
IL-6
TLR 7 agonist
Isotype- APC
Isotype-PE
TNFα
IFNα
IL-10
IL-6
TNFα
IFNα
IL-10
IL-6
Isotype- APC
Isotype-PE
TLR 9 agonist
Isotype- APC
Isotype-PE
Isotype- APC
Isotype-PE
TNFα
IFNα
IL-10
IL-6
TNFα
IFNα
IL-10
IL-6
Supplemental Figure 4: PBMCs from a subset of healthy controls and patients with active SLE disease were cultured in the absence or presence of TLR7 agonist (ORN R 2336) or TLR9 agonist (ODN2216) for 6 hours. Cells were then fixed and stained for intracellular levels of IFNα, TNFα, IL-6, and IL-10. Representative gating strategies are shown on pDCs population.
